# 1022. Evaluating the Impact of GenMark Dx ePlex® Blood Culture Identification (BCID) on Gram-negative Bloodstream Infections

**DOI:** 10.1093/ofid/ofab466.1216

**Published:** 2021-12-04

**Authors:** Pia Cumagun, Jeremy Meeder, Derek Moates, Hannah Pierce, Todd P McCarty, Rachael A Lee, Sixto M Leal

**Affiliations:** 1 University of Alabama at Birmingham, Birmingham, Alabama; 2 University of Alabama at Birmingham; Birmingham VA Medical Center, Birmingham, Alabama

## Abstract

**Background:**

The GenMark Dx ePlex BCID Gram-Negative (GN) panel utilizes electrowetting technology to detect the most common causes of GN bacteremia (21 targets) and 6 antimicrobial resistance (AMR) genes from positive blood culture (BC) bottles. Rapid detection of extended spectrum β-lactamases (ESBL: CTX-M & carbapenemases: KPC, NDM, IMP, VIM, OXA 23/48), and highly resistant bacteria such as *S. maltophilia* should enable early optimization of antimicrobial therapy.

**Methods:**

In this prospective study, aliquots of positive BC bottles with GN bacteria detected on Gram stain (GS) (n=108) received standard of care (SOC) culture and antimicrobial susceptibility testing (AST). Additionally, samples were evaluated with the BCID-GN panel but only SOC results were reported in the EMR and available to inform clinical decisions. Chart reviews were performed to evaluate the impact of the BCID-GN panel on the time to organism identification, AST results, and optimization of antimicrobial therapy.

**Results:**

A total of 108 patients are included in the analysis (Table 1). *Escherichia coli* was the most common bacteria identified followed by *Klebsiella pneumoniae, Pseudomonas aeruginosa,* and *Enterobacter* species (Table 2). There were 11 (10.2%) polymicrobial bacteremias. Repeat BCs were obtained in 68 (63%) patients of which 13 (19%) were persistently positive. Eight (7%) patients had evidence of additional gram-positive (GP) pathogens. Organism identification occurred 26.7 hours faster than culture. In conjunction with GS, negative pan-GP marker data could have helped providers make the decision to remove GP antibiotic coverage in 63 (58%) patients. Narrowing from empiric meropenem could have occurred in 5 patients. Of 10 individuals infected with resistant isolates (1 *S. maltophilia*, 1 OXA 23/48, and 8 CTX-M) empiric therapy was ineffective in 4 (40%) cases. Optimization of antimicrobial therapy for 9 (8.3%) patients could have occurred an average of 52.4 hours earlier than standard methods.

Table 1. Patient demographics and co-morbidities.

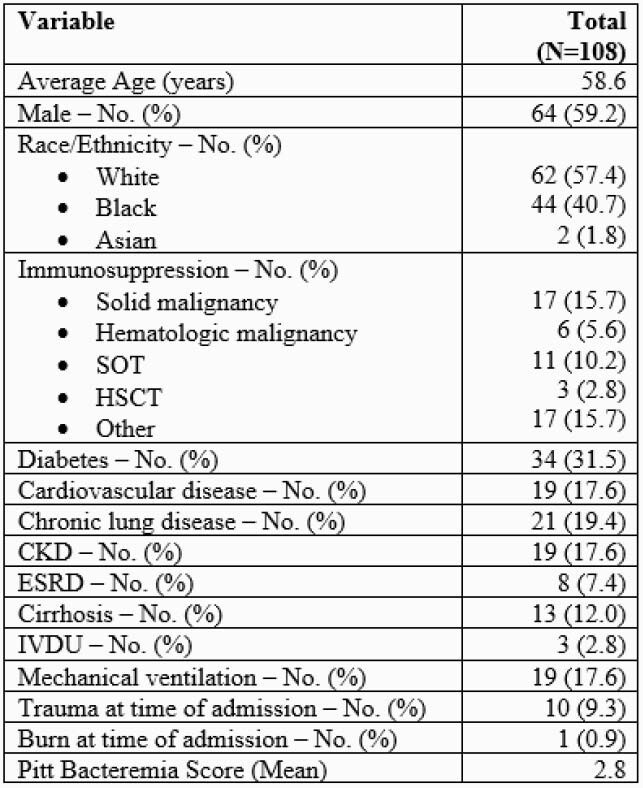

Table 2. Gram-negative bacteria frequency.

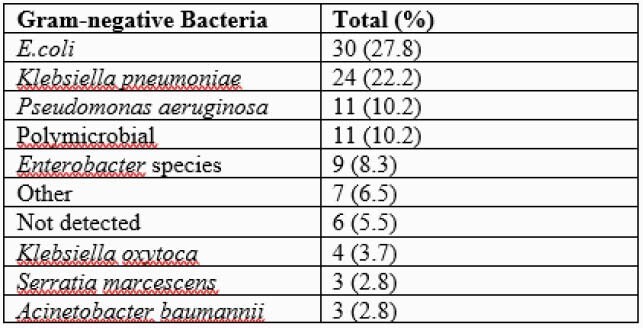

**Conclusion:**

The BCID-GN panel enabled earlier time to optimal treatment of highly resistant bacteria as well as multiple opportunities for narrowing gram negative spectrum and a higher degree of certainty in cessation of broad-spectrum gram-positive antibiotics

**Disclosures:**

**Todd P. McCarty, MD**, **Cidara** (Grant/Research Support)**GenMark** (Grant/Research Support, Other Financial or Material Support, Honoraria for Research Presentation)**T2 Biosystems** (Consultant) **Sixto M. Leal, Jr., MD, PhD**, **Abnova** (Grant/Research Support)**AltImmune** (Grant/Research Support)**Amplyx Pharmaceuticals** (Grant/Research Support)**Astellas Pharmaceuticals** (Grant/Research Support)**CNINE Dx** (Grant/Research Support)**GenMark Diagnostics** (Grant/Research Support, Other Financial or Material Support, Honoraria- Research Presentation)**IHMA** (Grant/Research Support)**IMMY Dx** (Grant/Research Support)**JMI/Sentry** (Grant/Research Support)**mFluiDx Dx** (Grant/Research Support)**SpeeDx Dx** (Grant/Research Support)**Tetraphase Pharmaceuticals** (Grant/Research Support)

